# Phenolics and Volatile Compounds of Fennel (*Foeniculum vulgare*) Seeds and Their Sprouts Prevent Oxidative DNA Damage and Ameliorates CCl_4_-Induced Hepatotoxicity and Oxidative Stress in Rats

**DOI:** 10.3390/antiox11122318

**Published:** 2022-11-23

**Authors:** Hassan Barakat, Ibrahim Ali Alkabeer, Thamer Aljutaily, Mona S. Almujaydil, Reham M. Algheshairy, Raghad M. Alhomaid, Abdulkarim S. Almutairi, Ahmed Mohamed

**Affiliations:** 1 Department of Food Science and Human Nutrition, College of Agriculture and Veterinary Medicine, Qassim University, Buraydah 51452, Saudi Arabia; 2 Food Technology Department, Faculty of Agriculture, Benha University, Moshtohor 13736, Egypt; 3 Department of Biochemistry, Faculty of Agriculture, Benha University, Moshtohor 13736, Egypt

**Keywords:** *Foeniculum vulgare*, sprouts, bioactive compounds, antioxidative efficiency, in vitro, in vivo

## Abstract

Researchers recently focused on studying the nutritional and functional qualities of sprouts generated from seeds. The current study investigated the total phenolic content (TPC), total flavonoids (TF), total flavonols (TFL), antioxidant activity (AOA), specific phenolic acids, and volatile chemicals in fennel seeds (FS) and fennel seed sprouts (FSS). The oxidative DNA damage prevention activity of selected FS and FSS extracts against DNA was examined. Consequently, the antioxidative stress potential of FS and FSS extracts at 300 and 600 mg kg^−1^ on CCl_4_-induced hepatotoxicity and oxidative stress in rats weas investigated. The liver’s functions and oxidative stress biomarkers in rat blood were examined. FSS exhibited rich phytochemical content such as TPC, TF, TFL, and AOA with altered phenolics and volatiles. HPLC identified nineteen compounds of phenolic acids and their derivatives in FS. Thirteen phenolics and six flavonoids were predominantly identified as Vanillic acid and Kaempferol, respectively. GC-MS analysis identified fifty and fifty-one components in FS and FSS, respectively. The predominant component was Benzene, [1-(2-propenyloxy)-3-butenyl] (*trans*-Anethole) (38.41%), followed by *trans*-Anethole (Benzene, 1-methoxy-4-(2-propenyl)) (23.65%), Fenchone (11.18%), and 1,7-Octadiene, 2-methyl-6-methylene- Cyclohexene (7.17%). Interestingly, α-Pinene, Fenchone, *trans*-Anethole (Benzene, 1-methoxy-4-(2-propenyl)), 4-Methoxybenzaldehyde (4-Anisaldehyde), Benzeneacetic acid, α-hydroxy-4-methoxy, and Nonacosane contents were increased. While Dillapiole, 7-Octadecenoic acid, and methyl ester were newly identified and quantified in FSS. The oxidative DNA damage prevention capability of FSS and FS extracts indicated remarkable DNA protection. Administrating FS and FSS extracts at 300 and 600 mg kg^−1^ ameliorated AST, ALT, and ALP, as well as GSH, CAT, MDA, and SOD, in a dose-dependent manner. The most efficient treatment of FS or FSS was using a dose of 600 mg Kg^−1^, which recorded an improvement rate of 20.77 and 24.17, 20.36 and 24.92, and 37.49 and 37.90% for ALT, AST, and ALP, respectively. While an improvement rate of 40.08 and 37.87%, 37.17 and 46.52%, 114.56 and 154.13%, and 66.05 and 69.69% for GSH, DMA, CAT, and SOD compared to the CCl_4_-group, respectively. The observed protection is associated with increased phenolics and volatiles in *F. vulgare*. Therefore, FS and FSS are recommended as functional foods with bioactive functionality, health-promoting properties, and desired prevention capabilities that may help prevent oxidative stress-related diseases.

## 1. Introduction

Oxidative stress is an imbalance between the incidence of reactive oxygen species and the detoxifying or repairing systems in the body [1]. Disturbance in the normal redox state of cells produces harmful peroxides and free radicals that destroy cell lipids, proteins, and DNA [2]. Oxidative stress from oxidative metabolism can break DNA and create underlying damage. The indirect base damage of reactive oxygen species (ROS) is caused by hydroxyl radicals, superoxide radicals, and hydrogen peroxide [2]. It can affect normal cellular signaling as some reactive oxidative species act as cellular messengers in redox signaling. In humans, oxidative stress is a probable cause of cancer, Alzheimer’s disease [1], atherosclerosis [3], and depression [4]. Researchers are looking for plant-based bioactive inhibitory agents against the effects of oxidative stress.

Sprouts are a phytonutrient-rich vegetable food that is a good source of proteins, minerals, vitamins, glucosinolates, isothiocyanates, flavonoids, and polyphenols [5]. Interestingly, according to Reed et al. [6], sprouts are considered “functional foods”, which are described as foods that offer additional health-promoting or disease-prevention advantages beyond their actual nutritional content. Studies on sprouts’ nutritional value, phytochemical composition during production or storage, and investigations into their microbiological, bioactive, and technological aspects have been indicated [7]. In the same context, the nutritional benefits and sensory acceptance of food products made with added sprouts were recently reviewed [8]. Researchers struggle to improve the nutritional value of seeds and make the most of the abundant supply of bioactive phytochemicals. Sprouting helps increase phenolic content, antioxidant capacity, glycemic index, and possible bioaccessibility [9]. Świeca et al. [10] confirmed that sprouting improved the nutraceutical value of lentil sprouts in terms of antioxidant capacity.

Fennel is one of the most common medicinal plants in the Apiaceae family (Umbelliferaceae) [11,12]. It demonstrates hepatoprotective, antioxidant, anti-thrombotic, anti-inflammatory, antibacterial, and antifungal qualities [13,14]. Antioxidant compounds (such as α-tocopherol, B complex, β-carotene, zinc salts, vitamins, glutamic acid, selenium salts, phosphor salts, hydrolyzed collagen, magnesium salts, chondroitin sulfate, soy lecithin, and glycosaminoglycan sulfate) have been shown to aid in peripheral nerve regeneration following injury [15]. A recent study found that expression levels of amyloid precursor protein isoforms and oxidative stress markers were stabilized by *F. vulgare*, reducing neuronal toxicity [16]. *F. vulgare* extract exhibits anti-cholinesterase activity and can be beneficial for recovering memory deficits in Alzheimer’s disease and dementia [17]. In addition, its stress-reducing properties allow for a corresponding improvement in memory. Antioxidant qualities may help reduce stress and conditions related to stress [18]. Moreover, central analgesic effects are bestowed by the methanolic extract of *F. vulgare* seeds, which also prevents the progression of inflammatory illnesses. *F. vulgare* is an exciting prospect for enhancing recovery from peripheral nerve injury due to its anti-inflammatory, analgesic, and antioxidative characteristics [19]. The phenolic components in fennel seeds promote healthy human growth, and organic extracts showed antibacterial activity against some human pathogenic microorganisms [20].

Essential oil from fennel seeds has potent antimicrobial and antioxidant properties [12,21,22,23]. In addition to these uses, fennel seeds’ essential oil has been shown to have pharmacological effects, including anti-inflammatory, antispasmodic, anti-thrombotic, laxative, anti-tumor, analgesic, antidiabetic, digestive, acaricidal, diuretic, nervous-disorder-curing, and hepatoprotective nature [12,23]. According to other studies, essential fennel oil is rich in chemicals, including α-pinene, fenchone, anethole, and estragol [12,21,22,23]. Fenchone is an aromatic mono-terpene [24] with a medicinal function for treating tuberculosis after some chemical modifications [25]. In addition, essential fennel oil has antifungal and acaricidal activity [26]. *Trans*-anethole is also an aromatic mono-terpene that exerts biological function, including anti-inflammation, neuroprotective effects, antinociceptive, anticonvulsant, anti-amnesic, and anti-cancer effects [27,28].

GC-MS analysis identified 31 component fractions, indicating 99.46% fennel seed extract. The majority was for α-Pinene, Fenchone, Estragole, (Z)-Anethole, 1,8-Cineole, Estragole, Anisaldehyde, and Carvacrol. In addition, eight main phenolic compounds recognized as antioxidant sources and their concentrations have been identified [29]. Alam et al. [30] reported that the GC-MS technique was used to identify fifty-seven distinct phytoconstituents in a methanolic extract of *F. vulgare*. The top chemicals were *trans*-anethole (31.49%), 2-pentanone (25.01%), fenchone (11.68%), and benzaldehyde-4-methoxy (8.01%). There were also substances found in relatively large quantities and others found at much lower levels. Among antioxidants, the extracts from *F. vulgare* seeds were found to be particularly effective at neutralizing free radicals while also displaying a wide range of bioactivity and practical utility. [14,15,29,30].

Until now, nobody has looked into the bio-changes in phytochemicals during FS sprouting, their oxidative DNA damage prevention activity, or their hepatoprotective efficiency. Therefore, phytochemicals and related antioxidant activity (AOA) were measured during sprouting. Selected FS and FSS extracts were examined for their ability to protect DNA from oxidative damage. In addition, phenolics were analyzed using high-performance liquid chromatography (HPLC), and volatiles using gas chromatography-mass spectrometry (GC-MS). Consequently, the antioxidative stress potential of FS and FSS extracts on CCl_4_-induced hepatotoxicity in an animal model was investigated.

## 2. Materials and Methods

### 2.1. Raw Fennel Seeds

Seeds of fennel (*F. vulgare*) were obtained from the Al-Tamimi market in the Qassim region of Saudi Arabia (https://www.tamimimarkets.com, accessed on 1 September 2020). A professor at the College of Agriculture and Veterinary Medicine at Qassim University in the Saudi Arabian Kingdom, Dr. Mokded Rabhi, confirmed the plant’s legitimacy. We discarded all the dust and broken and spoiled seeds. Green fennel sprouts were developed right away from clean seeds. Before being analyzed or sprouted, raw or milled seeds (American model laboratory mill, model ES2097) were stored at 4 ± 1 °C in plastic bags.

### 2.2. Sprouting of F. vulgare and Preparation of Aqueous and Ethanolic Extracts

One hundred grams of seeds were immersed in sodium hypochlorite solution before sprouting (1% for 3 min). Following a three-time rinsing in sterilized distilled water (sd.H_2_O), the seeds were equally placed on 7 × 25 cm^2^ plastic trays and germinated in a seed germinator (Easygreen, Canada). The seeds were germinated in a seed sprouter with a thermostat and an atomizer at 17 ± 1 °C with a relative humidity of 91%. Every day for the first three days, 20.0 mL sd.H_2_O/tray was used to spray the fennel seeds. From the first day of germination up until the 15th day, samples were taken at 3-day intervals. After being frozen at −18 ± 1 °C for a whole night, fennel sprouts were freeze-dried (CHRIST, Alpha 1-2 LD plus, Osterode, Germany) at −52 °C and 0.032 mbar for 96 h. After the freeze-dried sprouts were obtained, they were ground in a small laboratory mill (Thomas Wiley, St. Louis, MO, USA) and sieved (60 mesh sieve) to prepare a homogenous powder and stored in the dark containers at 4 ± 1 °C until the phytochemicals, HPLC, and GC-MS analyses were performed. To conduct the biological assessment of FS and FSS, 1 kg of FSS was separately germinated under the same conditions for 9 days, gradually dried by following a 24 h drying program according to Barakat et al. [31] and Al-Qabba et al. [32] milled, sieved, and kept in cold storage until extraction. Approximately one kg of FS or FSS was extracted three times with 5000 mL of 70% ethanol to prepare the ethanolic extracts (FS-EE and FSS-EE). One kg of FS or FSS was extracted three times with 5000 mL hot water (70 °C for 10 min) to prepare the aqueous extract (FS-AE or FSS-AE). All filtered extracts were concentrated by a rotary evaporator at 40 °C to evaporate the remaining solvent or water. The residues were frozen overnight, then freeze-dried for 96 h at –52 °C using (CHRIST, Alpha 1-2 LD plus, Osterode, Germany), and 0.032 mbar [33]. Freeze-dried samples were pulverized using a porcelain mortar and pestle to prepare a homogeneous powder that was kept in dark packages at 4 ± 1 °C until used.

### 2.3. Determination of Total Phenolic Content (TPC), Total Flavonoids (TF), and Total Flavonols (TFL) in FS and FSS

Folin-Ciocalteu reagent was used to measure the TPC of *F. vulgare* seeds and sprouts. In summary, a gram of freeze-dried FS or FSS samples was extracted in 10 mL of 70% methanol, the supernatant was collected, and the volume was adjusted up to 10 mL with the extraction solvent. An equal volume of Folin-Ciocalteu reagent (1:10) and aliquots of clear supernatant were mixed and incubated for 5 min before the reaction was stopped by adding Na_2_CO_3_ (7.5 %). The TPC content was calculated as milligrams of Gallic acid equivalents (GAE) per gram based on an OD reading at 765 nm taken 60 min after incubation and compared to a standard curve derived from Gallic acid (GA) solution (*R*^2^ = 0.99) (mg of GAE g^−1^ DW) [34]. The TF content of FS and FSS using the same methanolic extract was determined. Briefly, 1 mL of clear extract aliquots were combined with 1 mL of 2% AlCl_3_ and monitored for 60 min at 420 nm. TFL concentrations in FS and FSS were calculated by reacting methanolic extract aliquots with sodium acetate (5%). AlCl_3_ (2%) was added after 5 min, and the optical density (OD) was measured after 150 min at 440 nm, as described by Mohdaly et al. [35]. The TF and TFL concentrations were reported in milligrams of Quercetin-Equivalent (mg QE) per gram of dry weight (mg QE g^−1^).

### 2.4. Antioxidant Capacity Determination

According to Barakat and Rohn’s method [36], spectrophotometric measurements of the bleaching of DPPH radicals in a purple solution were used to determine the radical scavenging activity (DPPH-RSA) of FS and FSS. In brief, a gram of freeze-dried FS or FSS samples was extracted in 10 mL of 70% methanol, then the supernatant was collected, and the volume was adjusted up to 10 mL with the extraction solvent. A 0.1 mL aliquot from the clear supernatant of FS and FSS was mixed with 2.9 mL of DPPH solution and kept in the dark for 60 min. The absorbance was measured at 517 nm. A calibration curve using Trolox as an antioxidant standard was constructed by relating the percentage of DPPH-RSA. The antioxidant activity was reported as micromoles of Trolox-Equivalents per gram (µmol TE g^−1^).

### 2.5. Quantification of Phenolic Compounds in F. vulgare and Its Sprouts by HPLC-DAD

Using an HPLC system HP1100 (Agilent Technologies, Palo Alto, CA, USA) equipped with an autosampler, quaternary pump, and diode array detector (DAD, Hewlett Packard 1050), a column (Altima C18, 5 × 150 mm, 4.6 mm ID), and a guard column (Altima C18, 5 mm, Alltech, Nicholasville, KY, USA) were used to determine the phenolic content in FS, 6th day, and 9th day sprouts According to Kim et al. [37]. The gradient of acetic acid concentrations in the solvent system was A (acetic acid 2.5%), B (acetic acid 8%), and C (Acetonitrile). We separated the extracted samples at 25 °C by injecting 10 µL at a flow rate of 1 mL min^−1^. Peaks of phenolic compounds (g g^−1^) were identified by comparing their retention times and mass spectra with the machinery library and external standards. The employed external standards were purchased from Sigma-Aldrich, St. Louis, MO, USA.

### 2.6. Quantification of Volatile Components by GC-MS

For this GC-MS study, we used a Thermo Scientific Trace GC Ultra/ISQ Single Quadrupole MS equipped with a TG-5MS fused silica capillary column (30 m, 0.251 mm, 0.1 mm film thickness) that we utilized. The electron ionization system used for GC-MS detection had an ionization energy of 70 eV. The carrier gas was helium flowing at a rate of 1 mL min^−1^. The injector and MS transfer line temperatures were set at 280 °C. Starting with 50 °C, the oven was set to rise to 150 °C at a rate of 7 °C min^−1^ (hold for 2 min), then to 270 °C at a rate of 5 °C min^−1^ (hold for 2 min), and finally to 310 °C at a rate of 3.5 °C min^−1^ as the final temperature (hold for 10 min). Calculating the relative peak area as a percentage allowed us to probe the indicated components’ quantification. According to Odeh and Allaf [29], tentative identification of the compounds was accomplished by comparing their relative retention times and mass spectra with the GC-MS system’s NIST and WILLY library data.

### 2.7. Animals and Experimental Design

Wistar rats (48 adult males) weighing 190–200 g were used in the investigation. All experiments were approved by the Institutional Animal Ethics Committee (IAEC) of QU and KSA with an assigned number (21-09-01 on Thursday, 10 February 2022). The animals were kept in polypropylene cages at a constant temperature of 24 ± 1 °C and 40–45% relative humidity in a controlled laboratory environment. Following a week of acclimation, rats were randomly divided into six groups (8 rats each). Rats were given identification tags, and their BW was recorded. The rats could access a standard pellet diet and water [38]. The following procedures were used on the various rat groups: Group 1 (normal rats, NR) was given olive oil (1.0 mL kg^−1^ twice weekly) through intraperitoneal injection (i.p.) and saline buffer (2 mL day^−1^ orally) for 6 weeks. To cause hepatotoxicity in rats used in experiments. An i.p injection of a fresh mixture of CCl_4_ and olive oil (at a concentration of 1.0 mL kg^−1^ twice weekly) and 2 mL of saline buffer were given orally/daily, then marked as Group 2 [39]. Group 3 received 300 mg kg^−1^ of FS-AE orally/daily along with CCl_4_ i.p twice a week. Group 4: received 600 mg kg^−1^ of FS-AE orally/daily along with CCl_4_ i.p twice a week. Group 5 received 300 mg kg^−1^ of FSS-EE orally/daily along with CCl_4_ i.p twice a week. Group 6 received 600 mg kg^−1^ of FSS-EE orally/daily along with CCl_4_ i.p twice a week. At the end of the 6th week, animals fasted for 12 h with free access to water. Rats were anesthetized with a mixture of Alcohol: Chloroform: Ether (1:2:3), according to Leila et al. [40], and then sacrificed. Fresh liver tissue was taken, washed three times with buffer phosphate buffer, and transferred into DNA-RNA-free tubes for DNA extraction following the protocol of Arseneau et al. [41]. Blood samples were collected from the heart punctures of all the animals. Blood tubes were subjected to serum separation for various biochemical parameters by centrifugation at 3000× *g* for 30 min under cooling. The biochemical parameters were determined using suitable kits and a blood chemistry analyzer (HumaLyzer 4000, HUMAN Gesellschaft für Biochemica und Diagnostica mbH, Max-Planck-Ring 21, 65205 Wiesbaden, Germany).

#### 2.7.1. Protective Effect of FS and FSS Extracts against H_2_O_2_-Induced DNA Damage

To study the protective effects of selected FS and FSS extracts against DNA damage induced by the Fenton reaction, the reaction was conducted in a microcentrifuge tube at a total volume of 15 μL containing 0.5 μg of rat liver DNA, 3 μL of 50 mM phosphate buffer (pH 7.4), 3 μL of 2 mM FeSO_4_, and 2 μL of FS or FSS at 0.5–5 mg mL^−1^ concentrations. Then, 4 μL of 30% H_2_O_2_ were added, and the mixture was incubated at 37 °C for 1 h [42]. Finally, the mixture was subjected to 1% agarose gel electrophoresis for 35 min, stained with ethidium bromide, visualized under a UV illuminator, and then captured using an Olympus camera. The DNA amounts were calculated by measuring the DNA-Band intensity using Kodak ID program v., 3.6 and the mean of three replicates ± SE was calculated and performed in Excel. A standard DNA curve was plotted between known DNA concentrations and their band intensity data (*R*^2^ = 0.953), and results were given in ng per band based on loading 8 µL for each reaction.

#### 2.7.2. Determination of Liver’s Functions

The liver’s functions such as alanine aminotransferase (ALT, UL^−1^), aspartate Aminotransferase (AST, UL^−1^), alkaline phosphatase (ALP, UL^−1^), and total bilirubin (T. Bili, mg dL^−1^) in blood serum were measured using an alanine aminotransferase kit (EC 2.6.1.2), an aspartate aminotransferase kit (EC 2.6.1.1), an optimum alkaline kit (EC 3.1.3.1), and a photometric test kit, respectively. All biochemical examination kits were purchased from Human Co., Wiesbaden, Germany.

#### 2.7.3. Oxidative Stress Biomarkers

According to the technique outlined by Beutler et al. [43], reduced glutathione (GSH, g dL^−1^) was determined using a GSH colorimetric test kit (E-BC-K030-S, Elabscience, Houston, TX, USA). According to Ohkawa et al. [44], lipid peroxidation was evaluated using a malondialdehyde (MDA, nmol mL^−1^) colorimetric assay kit (E-BC-K025-S, Elabscience, Houston, TX, USA) by detecting the thiobarbituric acid reactive substance (TBARS) MDA complex. The absorbance of the generated colored complex was measured at 532 nm and calculated as nmol mL^−1^. Giannopolitis and Ries’ method [45] was used to measure the activity of superoxide dismutase (SOD, U L^−1^) using a SOD-type activity assay kit (E-BC-K022-S, Elabscience, Houston, TX, USA). The color reaction was measured at 550 nm, expressed as U L^−1^. Utilizing a CAT activity test kit (E-BC-K031-S, Elabscience, Houston, TX, USA), the catalase (CAT, U L^−1^) activity was assessed using the method of Aebi [46]. All oxidative stress markers were determined using a blood chemistry analyzer (HumaLyzer 4000, HUMAN Gesellschaft für Biochemica und Diagnostica mbH, Max-Planck-Ring 21, 65205 Wiesbaden, Germany).

### 2.8. Statistical Analysis

SPSS was used for the statistical analysis (Ver. 22.0 for Windows, IBM, Chicago, IL, USA). All experimental data was reported as a mean ± SE. According to Steel et al. [47], statistical significance was determined using one-way ANOVA followed by post hoc testing, and *p*-values < 0.05 were applied.

## 3. Results

### 3.1. Phytochemicals and Antioxidant Activity of F. vulgare Sprouts

**Figure 1:**
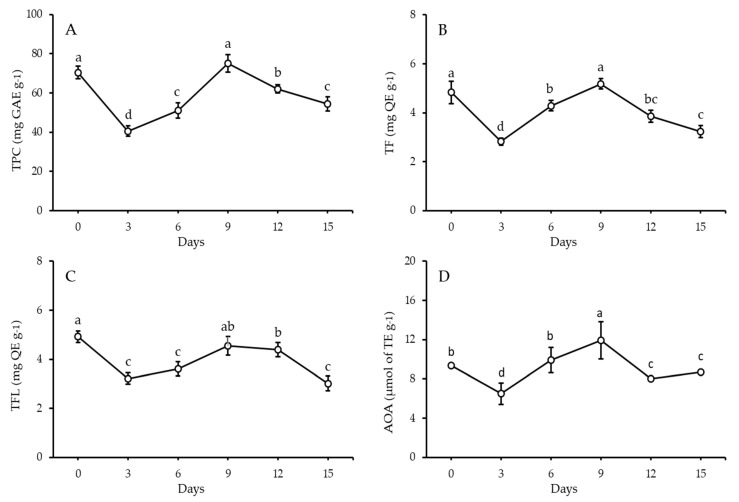
Total phenolic content (**A**), total flavonoids (**B**), total flavonols (**C**), and potential antioxidant activity (**D**) of FS after 15 days of sprouting at 17 ± 1 °C and 90–93% RH (mean ± SE), *n* = 6. ^a,b,c,d^: There is a statistically significant (*p* > 0.05) difference between bars that do not share identical lettering.

Quantitative analysis of phytochemicals such as TPC, TF, and TFL and related antioxidant activity using DPPH radical scavenging (AOA) in FS and FSS at 3, 6, 9, 12, and 15 days was performed. The TPC content of FS was 70.42 mg GAE g^−1^, as demonstrated in Figure 1. Both TF and TFL contents in FS were 4.83 and 4.93 mg QE g^−1^, respectively. Antioxidant activity levels were tracked over time using the DPPH-RSA assay. The results showed 9.36 µmol of TE g^−1^ in FS. On the 3rd and 6th days, significant decreases in TPC, TF, TFL, and AOA were observed. On the contrary, the TPC, TF, TFL, and AOA content exhibited significant increases to be the highest values during the sprouting period, indicating a nonsignificant difference compared to FS. Obviously, the contents of TPC, TF, TFL, and AOA gradually decreased with the sprouting period’s progression. Further, the FS and both the 6th and 9th day sprouts were selected to start building new components and to analyze individual phenolics and volatiles using HPLC and GC-MS, respectively.

### 3.2. Quantification of Phenolic Compounds in FS and FSS

Extracts from FS sprouts, 6-day sprouts, and 9-day sprouts were analyzed quantitatively for phenolics, and the results are shown in Table 1 and Supplementary Figure S1. FS and its sprouts contained measurable levels of 13 different phenolic acids and 6 different flavonoids. Vanillic acid (587.40 µg g^−1^) was the most abundant phenolic, followed by O-Coumaric acid (112.77 µg g^−1^) and Rosmarinic acid (64.41 µg g^−1^). According to Table 1, the FS has a high concentration of flavonoids. The highest concentrations of flavonoids were found to be Kaempferol (5913.55 µg g^−1^), Resveratrol (472.19 µg g^−1^), and Rutin (423.28 µg g^−1^), followed by Myricetin (236.93 µg g^−1^), Catechin (123.46 µg g^−1^), and Quercetin (28.71 µg g^−1^).

A rise in phenolics was seen on the sixth day of sprouting; these included Catechol, *p*-Hydroxy benzoic acid, Chlorogenic acid, Cinnamic acid, Ellagic acid, Ferulic acid, *p*-coumaric acid, Benzoic acid, and Syringic acid. On the other hand, phenolics, including Caffeic acid, Vanillic acid, and Rosmarinic acid, were reduced. Consequently, flavonoids such as Catechin, Quercetin, and Rutin increased, while a dramatic decrease in Kaempferol, Myricetin, and Resveratrol was observed.

On the 9th day of sprouting, the most abundant phenolic acid was Vanillic acid which was retained only by 22%. A reduction in *O*-Coumaric acid by 92.52% was recorded. Interestingly, Catechol, *p*-Hydroxy Benzoic acid, Caffeic acid, Chlorogenic acid, Cinnamic acid, Ellagic acid, Ferulic acid, *p*-Coumaric acid, Benzoic acid, Rosmarinic acid, and Syringic acid contents were increased 7.80, 1.01, 1.80, 10.49, 2.01, 2.06, 2.42, 1.08, 3.63, 1.94, and 6.8-fold, respectively. In the same context, Catechin, Quercetin, and Rutin were increased, whereas all flavonoids were decreased on the 9th day of sprouting.

### 3.3. Identification and Quantification of Volatiles in FS and FSS by GC-MS

**Figure 2:**
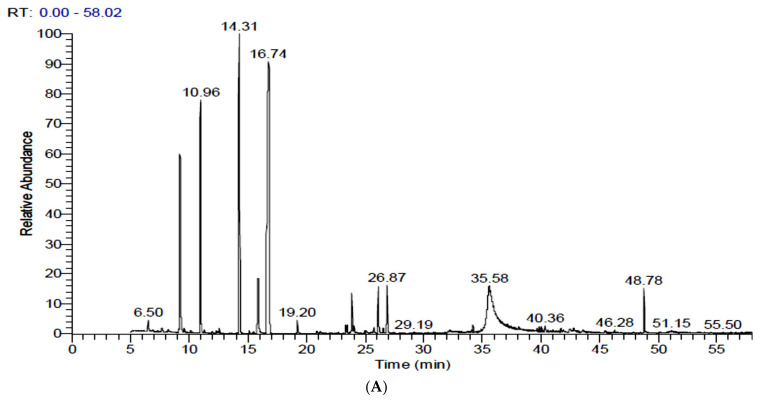

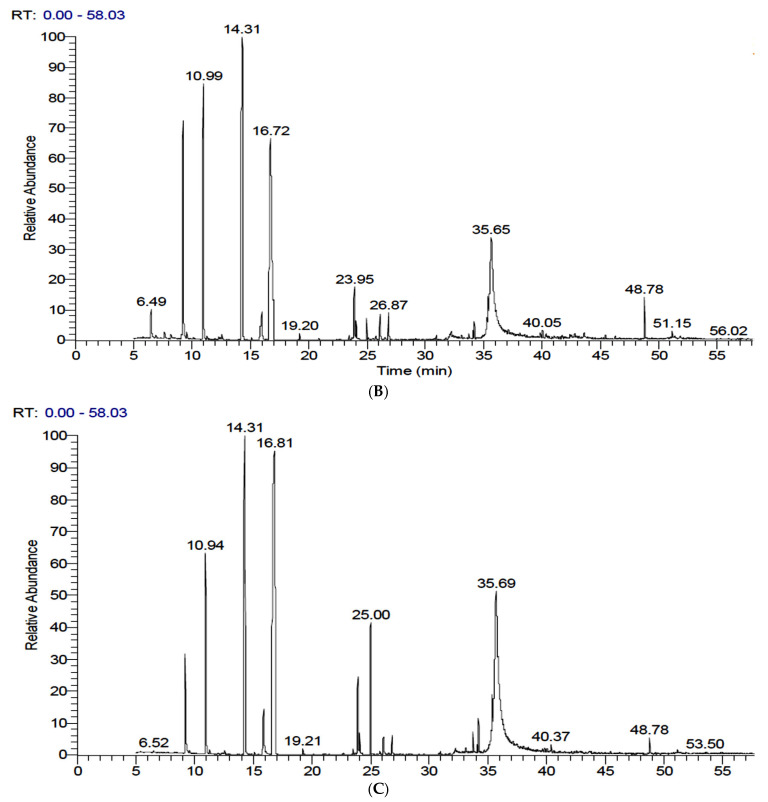
GC-MS chromatograms of fennel seeds and their sprouts after 15 days of sprouting at 17 ± 1 °C and 90–93% RH. (**A**): Raw fennel seeds, (**B**): 6-day sprouts, and (**C**): 9-day sprouts.

Table 2 and Figure 2 show the identification and concentration (%) of volatile components in extracts of FS and FSS. Fifty and fifty-one components were identified in FS and FSS, respectively (complete data was not shown; only concentrations greater than 1% have been presented). The GC-MS analysis of FS exhibited eleven components at a concentration higher than 1%. However, the predominant component was Benzene, [1-(2-propenyloxy)-3-butenyl] (*trans*-Anethole) (38.41%), followed by *trans*-Anethole (Benzene, 1-methoxy-4-(2-propenyl)) (23.65%), Fenchone (11.18%), and 1,7-Octadiene, 2-methyl-6-methylene- Cyclohexene (7.17%). GC-MS analysis of FSS resulted in newly synthesized components in addition to changes in the content of predominant components. Interestingly, α-Pinene, Fenchone, *trans*-Anethole (Benzene, 1-methoxy-4-(2-propenyl)), 4-Methoxybenzaldehyde (4-Anisaldehyde), Benzeneacetic acid, α-hydroxy-4-methoxy, and Nonacosane contents were increased at FSS after 6-days. In contrast, 4-Methoxybenzaldehyde (4-Anisaldehyde), Benzene, [1-(2-propenyloxy)-3-butenyl] (*trans*-Anethole), Benzeneacetic acid, and α-hydroxy-4-methoxy contents were increased at FSS after 9-days. On the contrary, 1,7-Octadiene, 2-methyl-6-methylene- Cyclohexene, 4,4′-Di(3-butenyl)-2,2′-bipyridine, Ethanone, 2-hydroxy-1,2-bis(4-methoxyphenyl), Ethanone, 2-hydroxy-1,2-bis(4-methoxyphenyl), 2-Diisobutylcarbamoyl-cyclohexane carboxylic acid, decyl ester and {[3E)-2-[(Dimethylcarbamoyl)methyl]-3-ethylidene-13,17-bis[2′(methoxyca bonyl)ethyl]2,7,12,18-tetramethyl-2,3dihydroporphytinato]}zinc (II) contents were decreased at FSS after 6 days. While, Dillapiole and 7-Octadecenoic acid, methyl ester was newly identified and quantified. Comparing the GC-MS compounds after 6 and 9 days of sprouting shows that the meaningful sprouting period for FS is 6 days. Unfortunately, some volatile compounds did not exist after sprouting, while other volatile compounds existed. Although some volatile compounds change the chemical formula through rearrangement and elongation for carbon chan or derivatives, all that is caused by physiological reactions during sprouting needs further study.

### 3.4. Protective Effect of FS and FSS Extracts on DNA Damage

**Figure 3:**
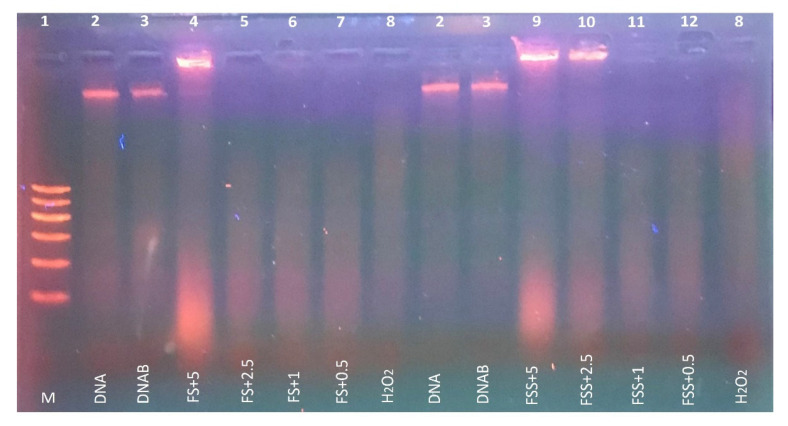
Protective effect of FS and FSS extracts at 2.5–5 mg mL^−1^ concentrations against H_2_O_2_-induced DNA oxidative damage in rat liver. Lane 1 (M): DNA marker, lane 2 (DNA): 0.5 μg rat liver DNA, lane 3 (DNAB): 0.5 μg rat liver DNA + 50 mM phosphate buffer, lane 4 (FS + 5): 0.5 μg DNA + 2 mM FeSO_4_ + 30% H_2_O_2_ + 50 mM phosphate buffer + FS extract (5 mg mL^−1^), lane 5 (FS + 2.5): 0.5 μg DNA + 2 mM FeSO_4_ + 30% H_2_O_2_ + 50 mM phosphate buffer + FS extract (2.5 mg mL^−1^), lane 6 (FS + 1): 0.5 μg DNA + 2 mM FeSO_4_ + 30% H_2_O_2_ + 50 mM phosphate buffer + FS extract (1 mg mL^−1^), lane 7: 0.5 μg DNA + 2 mM FeSO_4_ + 30%H_2_O_2_ + 50 mM phosphate buffer + FS extract (0.5 mg mL^−1^), lane 8 (H_2_O_2_): 0.5 μg DNA + 2 mM FeSO_4_ + 30% H_2_O_2_ + 50 mM phosphate buffer, lane 9 (FSS + 5): 0.5 μg DNA + 2 mM FeSO_4_ + 30% H_2_O_2_ + 50 mM phosphate buffer + FSS extract (5 mg mL^−1^), lane 10 (FSS + 2.5): 0.5 μg DNA + 2 mM FeSO_4_ + 30% H_2_O_2_ + 50 mM phosphate buffer + FSS extract (2.5 mg mL^−1^), lane 11 (FSS + 1): 0.5 μg DNA + 2 mM FeSO_4_ + 30% H_2_O_2_ + 50 mM phosphate buffer + FSS extract (1 mg mL^−1^), and lane 12: 0.5 μg DNA + 2 mM FeSO_4_ + 30% H_2_ O_2_ + 50 mM phosphate buffer + FSS extract (0.5 mg mL^−1^).

The protective effect of selected FS and FSS extracts at a concentration of 0.5, 1, 2.5, and 5 mg mL^−1^ against H_2_O_2_-induced DNA damage in rat liver DNA is presented in Figure 3. The Fenton reaction generates hydroxyl radicals, which induce DNA strand breaks in rat liver DNA. The presence of H_2_O_2_ and ferrous sulfate leads to severe DNA damage (Lane 8). FS extract at 5 mg mL^−1^ (Lane 4) and FSS extract at 2.5–5 mg mL^−1^ (Lanes 9 and 10) showed superior protection against DNA damage induced by hydroxyl radicals as compared to untreated DNA (lane 2) or mixed DNA with buffer phosphate 7.4 (lane 3) in rat liver DNA. The DNA concentration in the band of lane 4, 9 and 10 was 389 ± 23, 427 ± 17, and 264 ± 29 ng, respectively. Comparing bands in lane 4 and lane 9, we noticed that FSS at 5 mg mL^−1^ protected the DNA by 110% compared to FS at the same concentration. Thus, our results indicated that FSS extract (Lanes 9) showed better protection than FS extract (Lane 4) against DNA damage, confirming that the antioxidant content in FSS extract is higher than in FS extract. The lower concentrations (0.5–2.5 mg mL^−1^) of FS and (0.5–1.0 mg mL^−1^) of FSS extracts could not show any visible protection against DNA damage.

### 3.5. The Liver’s Functions

**Figure 4:**
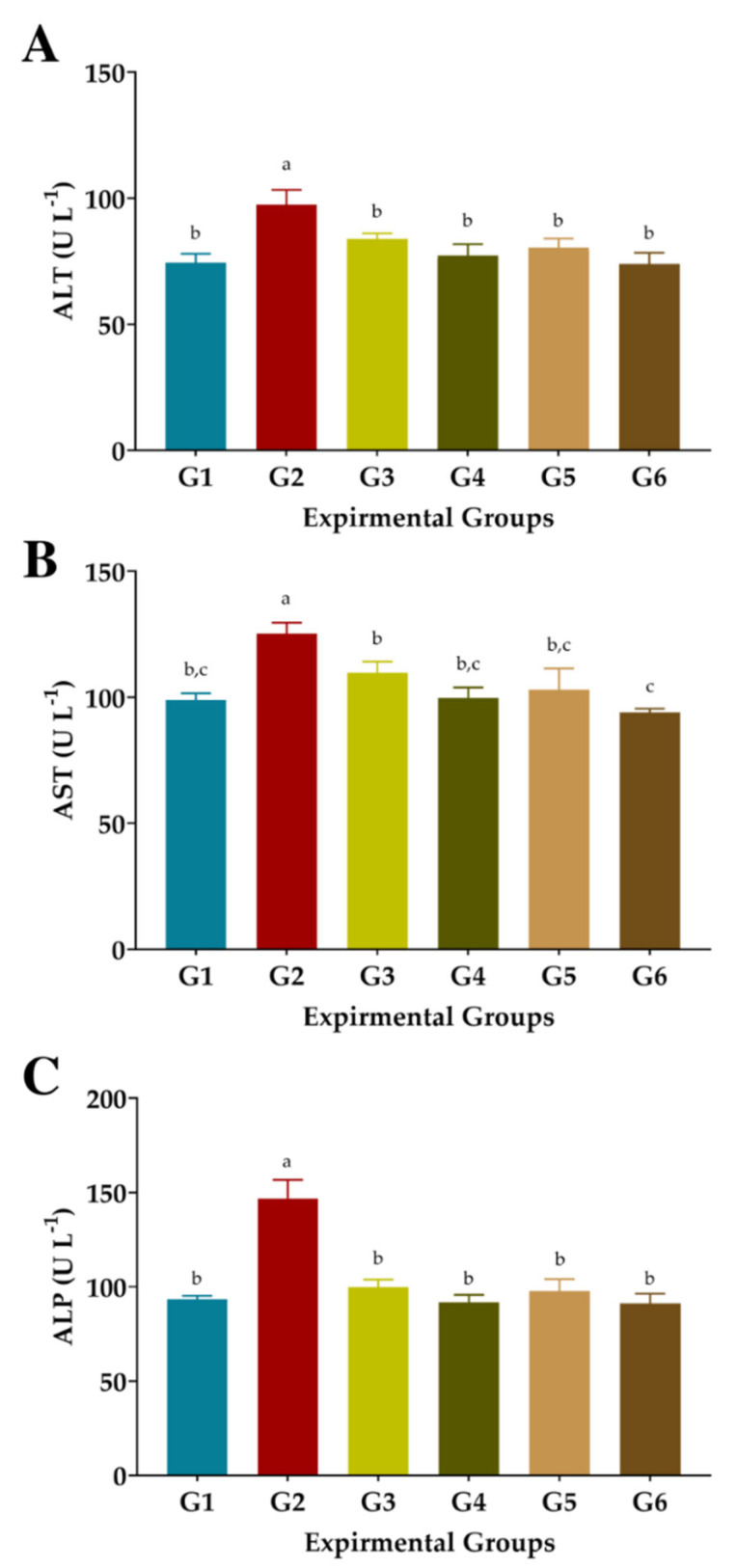
Effect of aqueous and ethanolic extracts of FS and FSS on liver functions in CCl_4_-induced oxidative stress and hepatotoxicity in rats (mean ± SE), *n* = 8. (**A**), ALT: Alanine aminotransferase, (**B**), AST: Aspartate aminotransferase, (**C**), ALP: Alkaline phosphatase, G1–G6: G1 (normal rats), G2 (positive control, CCl_4_-injected), G3 (CCl_4_-injected and received 300 mg kg^−1^ of FS-AE orally). G4 (CCl_4_-injected and received 600 mg kg^−1^ of FS-AE orally), G5 (CCl_4_-injected and received 300 mg kg^−1^ of FSS-EE orally), and G6 (CCl_4_-injected and received 600 mg kg^−1^ of FSS-EE orally), ^a,b,c^: Bars not sharing similar letters are significantly different (*p* > 0.05).

CCl_4_ injection substantially raised serum ALT, AST, and ALP enzyme levels in rats (G2) as oxidative stress and hepatotoxicity complications compared to normal rats (GI), (Figure 4). Administration of FS or FSS at 300 or 600 mg kg^−1^ improved the liver’s function and attenuated the liver’s enzyme changes. Administration of FS or FSS at a high level was better than using a low level to improve liver functions, regardless of the type of extract. Interestingly, giving FS and FSS reduced the alterations in liver functions caused by CCl_4_ injection to be close to normal values in GI (Figure 4A–C). The ALT level attenuated by 14.02, 20.77, 17.53, and 24.17% when 300 and 600 mg kg^−1^ of FS and FSS were given, respectively. Similarly, AST and ALP improved by 12.34, 20.36, 17.72, and 24.92% and 32.03, 37.49, 33.45, and 37.90%, respectively. However, FS and FSS markedly improved the liver enzymes (as presented in ALT, AST, and ALP) in a type- and dose-dependent manner compared to normal rats in G1.

### 3.6. Antioxidant Biomarkers

**Figure 5:**
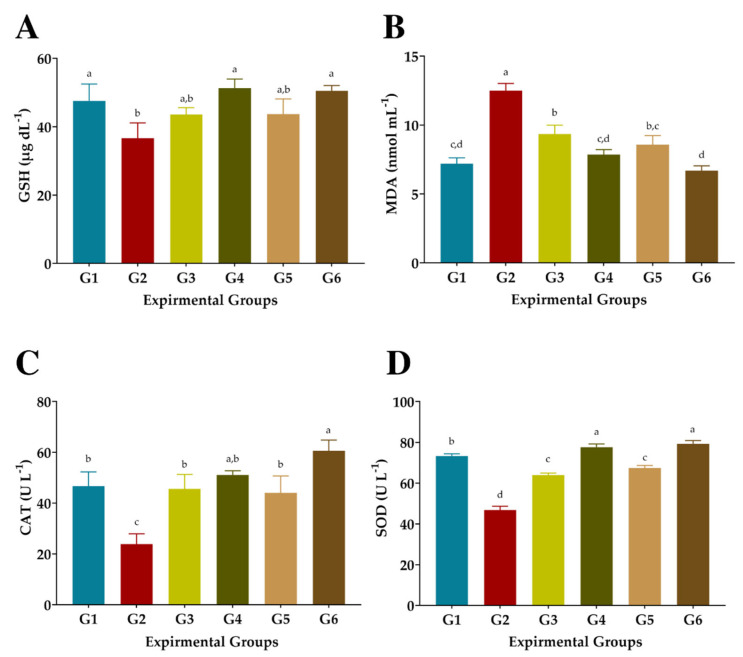
Effects of aqueous and ethanolic extracts of FS and FSS on antioxidant biomarkers in CCl_4_-induced oxidative stress and hepatotoxicity in rats (mean ± SE), *n* = 8. (**A**) GSH: Reduced glutathione, (**B**) MDA: Malondialdehyde, (**C**) CAT: Catalase, and (**D**) SOD: Superoxide dismutase, G1-G6: G1 (normal rats), G2 (positive control, CCl_4_-injected), G3 (CCl_4_-injected and received 300 mg kg^−1^ of FS-AE orally). G4 (CCl_4_-injected and received 600 mg kg^−1^ of FS-AE orally), G5 (CCl_4_-injected and received 300 mg kg^−1^ of FSS-EE orally), and G6 (CCl_4_-injected and received 600 mg kg^−1^ of FSS-EE orally), ^a,b,c,d^: Bars not sharing similar letters are significantly different (*p* > 0.05).

As shown in Figure 5, injection of CCl_4_ significantly reduced GSH, CAT, and SOD levels and increased the MDA level in the blood serum of G2 compared to normal rats in G1. Treated rats with FS or FSS at 300 or 600 mg kg^−1^ presented significant improvements in the activities of the antioxidant enzymes GSH, CAT, and SOD, as well as a substantial reduction in MDA levels (Figure 5). However, administration of 300 mg kg^−1^ FS or FSS caused moderate attenuation in GSH, CAT, and SOD and combated the autoxidation process, resulting in low MDA levels. The most efficient treatment of FS or FSS was using a dose of 600 mg kg^−1,^ which recorded an improvement rate of 40.08% and 37.87%, 37.17%, and 46.52%, 114.56 and 154.13, and 66.05 and 69.69% for GSH, DMA, CAT, and SOD when compared to the CCl_4_-group (G2), respectively. Interestingly, treating rats with 600 mg Kg^−1^ FSS extract enhanced the enzymatic defense system significantly better than FS extract compared to normal rats (G1) and CCl_4_-treated rats (G2).

## 4. Discussion

Antioxidant potential, regulation of blood pressure, interaction with gut microbiota, suppression of pro-inflammatory cytokine overproduction, and activation of antioxidant enzymes are only some of the mechanisms by which functional foods have been demonstrated to aid in disease control [32,48,49]. Biologically active substances like phenolic compounds have been lauded for their ability to combat metabolic diseases and as effective antioxidant substances, neutralizing free radicals including hydrogen peroxide, hydroxyl radicals, and superoxide anion [50]. When phenolics are incorporated, it’s been regarded as a promising strategy [51] with superior antioxidant activity. Higher phenolic component concentrations correlate with greater antioxidant power [32,52,53,54]. However, the current study aims to provide primary research that might be used as a foundation for producing fennel sprouts by providing valuable data on the bio-changing and fate of active molecules found in fennel sprouts as polyphenols and volatiles. Fennel seeds are feasible to cultivate, and the results of the current study suggest that sprouts may represent a novel source of active chemicals with superior antioxidants [55].

Interestingly, during sprouting, phenolics and antioxidants increased [10]. Unfortunately, the washing and soaking steps drastically decreased the TPC and related AOA at the beginning of the sprouting process. Leaching of such compounds (i.e., soluble proteins, water-soluble antioxidants, phytic acid, and tannins) may occur due to osmotic pressure causing the immigration of some bioactive compounds. As previously mentioned, this action affected the TPC and related AOA [56]. Consequently, our results exhibited a rise in TPC up to day 6 by 1.26 fold, indicating the synthesis of new bioactive compounds. This finding was supported by an increase in the AOA by 1.52 fold between days 3 and 6. With the progression of the sprouting period, a substantial increase in TPC and AOA was noticed. It is shown that newly generated TPC increased the AOA, which possesses antioxidative and ameliorative efficiency, as confirmed by Al-Qabba et al. [32,57,58]. In parallel, new flavonoids and flavanols have been generated during sprouting [59]. Our results were in harmony with Salama et al. [60], who recorded that TF content was in the range of 4.03–6.96 mg QE g^−1^ fennel seeds and agreed with Anwar et al. [61], while it was lower than remarked by Faudale et al. [62]. For TFL, the results of Salama et al. [60] exhibited a content in a range of 2.0–4.89 mg QE g^−1,^ which was agreed upon by Anwar et al. [61] and was lower than Faudale et al. [62] and higher than Ferioli et al. [63]. However, it should be mentioned that the number and content of phenolic compounds in seeds may be strongly influenced by genotype (species/variety), soil, environmental conditions, maturity level at harvest, post-harvest storage conditions, and extraction conditions [64]. Consumption of sprout extracts could help reduce cellular oxidation, as confirmed in the current study [32,65]. The total antioxidant activity (TAA) method, the DPPH scavenging activity assay, and the reducing power assay all demonstrated that the phenolic compounds present in the preparations possessed antioxidant and radical-scavenging characteristics [54]. The increased number of phenolics in *F. vulgare* sprouts increased more than its seeds with the progression of the sprouting period, which corroborated the findings of Swieca and Gawlik-Dziki [10]. *F. vulgare* seeds and sprouts show superior flavonoid content, similarly presented in many plants [66].

HPLC analysis concluded that thirteen phenolic compounds were identified, with vanillic acid as the predominant acid. Six flavonoids were also identified, Kaempferol being the most abundant. These results differed from those of Odeh and Allaf [29], who found that Vanillic acid is the highest phenolic compound but agreed when they discovered that the third most abundant phenolic acid is Rosmarinic acid. In the same context, Roby et al. [67] indicated lower rosmarinic acid (14.99 μg) than presented in our study. A difference in phenolic compound levels among raw fennel seeds and evident changes in flavonoids and phenolic acids during sprouting were observed. The content of each phenolic compound of fennel seeds and their sprouts at 0, 6, and 9 days was monitored. For example, vanillic acid was recorded as the predominant acid, whereas it was rarely identified in other studies [60,62,63]. Remarkably, Caffeic acid, *p*-coumaric acid, and Rosmarinic acid content decreased in 6-day sprouts and then increased by 80%, 7%, and 93% in 9-day sprouts, respectively. Similarly, the Ferulic acid content of 9-day sprouts increased from 20.01 μg g^−1^ to 48.51 μg g^−1^. The results were higher than those obtained by Odeh and Allaf [29], who found that the ferulic acid content in fennel seeds was 2.31 μg g^−1^. The observed increases in phenolics may be due to the sprouting process improving the degradation and extraction of phenolic acids and phenolic compound synthesis [68]. Unfortunately, no studies have confirmed the bio-changes in fennel seeds during sprouting. Generally, the change in phenolic profile with increasing and decreasing phenolic acid content was confirmed [32,64]. Six flavonoid compounds (Kaempferol, Resveratrol, Quercetin, Catechin, Myricetin, and Rutin) were identified and estimated, with the highest being Kaempferol and Resveratrol and the lowest being Quercetin. The results were higher than those obtained by Mohamad et al. [69], where the Kaempferol content was negligible. Also, the results were higher than those obtained by Allaithy [70] for catechin (17.36 μg g^−1^) and quercetin (5.30 μg g^−1^). Among the twenty-four chemicals found in fennel seeds, Castaldo et al. [55] identified five compounds (*p*-coumaric, ferulic, caffeic, chlorogenic acids, and quercetin).

The GC-MS analysis resulted in fifty and fifty-one components in FS and FSS, respectively. The GC-MS analysis of FS exhibited eleven components at a concentration higher than 1%. However, the predominant component was Benzene, [1-(2-propenyloxy)-3-butenyl] (*trans*-Anethole) (38.41%), followed by *trans*-Anethole (Benzene, 1-methoxy-4-(2-propenyl)) (23.65%), Fenchone (11.18%), and 1,7-Octadiene, 2-methyl-6-methylene- Cyclohexene (7.17%). Our results were nearly identical to those of other researchers for the main components but a little different in amounts [12,23]. It was also closely indicated by Suleiman and Helal [71], who identified fifty-seven different phytoconstituents in the methanolic extract of *F. vulgare* using the GC–MS technique. The main compounds identified were trans-anethole (31.49%), 2-pentanone (25.01%), fenchone (11.68%), and benzaldehyde-4- methoxy (8.01%). Intriguingly, after 6 days, α-pinene, Fenchone, *trans*-Anethole (Benzene, 1-methoxy-4-(2-propenyl)), 4-methylbenzaldehyde (4-anisaldehyde), Benzene acetic acid, -hydroxy-4-methoxy, and Nonacosane concentrations were increased. Consequently, sprouting for 9 days led to elevated levels of 4-Methoxybenzaldehyde (4-Anisaldehyde), Benzene, [1-(2-propenyloxy)-3-butenyl] (*trans*-Anethole), Benzeneacetic acid, and α-hydroxy-4-methoxy in FSS. Interestingly, this study reports the discovery and quantification of Dillapiole, 7-octadecenoic acid, and methyl ester newly found. The GC-MS analysis of 6- and 9-day sprouts indicated that the optimal sprouting time for FS is between 6 and 9 days. Unfortunately, some volatile chemicals’ levels dropped after sprouting while others’ levels remained stable. A recent study by Ilardi et al. [72] showed different profiles in different fennel parties as the main components of the roots were terpinolene (33.15%), γ-terpinene (12.18%), and fenchyl acetate (11.23%). Stems and leaves were very rich in α-phellandrene (36.85% and 41.59%, respectively) and β-phellandrene (19.68% and 25.79%, respectively), whereas the main components of fruits were terpinolene (20.10%) and limonene (17.84%). Also, Hong et al. [73] indicated that *trans*-anethole and fenchone had the highest and second-highest concentrations among fennel essential oil volatiles. To our knowledge, there has been no research on the bio-changes that occur in phyto-constitutes and volatiles during FS sprouting. Further research is required to determine the full view of the physiological responses triggered by sprouting, even though some volatile substances alter the chemical formula, rearrangement, and elongation of carbon chan or derivatives. For this reason, the present study can be seen as a springboard for future research.

Evidently, the quantification of phenolics and volatiles in *F. vulgare* sprouts increased dramatically with prolonged sprouting time [9,10,32], indicating benefits in biology and nutrition [7,8]. Biologically active substances like phenolic compounds have been lauded for their ability to combat metabolic diseases and as effective antioxidant substances, neutralizing free radicals including hydrogen peroxide, hydroxyl radicals, and superoxide anion [74]. In the present investigation, we found that compared to FS extract, FSS extract was more effective in preventing oxidative DNA damage, which could be related to increased bioactive components after sprouting [35,75]. Nonetheless, both FS and FSS, when present in sufficiently high quantities, have markedly greater antioxidative efficacy against generated radicals. Similarly, antioxidant protection in calf thymus DNA has been demonstrated by using seed extracts. These extracts have been proven to be resistant to the harmful effects of several diseases, including cancer, atherosclerosis, diabetes, inflammation, and aging [75]. Fennel and clove oils synergized to cause apoptosis in Caco-2 cells through S and G2/M phase arrest [72]. According to El-Garawani et al. [76], fennel oil may protect against etoposide-induced genocytotoxicity in male albino rats. Therefore, the present data imply that ingesting FS and FSS could be more effective in reducing DNA damage, which may protect against diseases such as liver, kidney, diabetes, and immune system diseases, as Hanan et al. [77] hypothesized.

In the second phase of this study, FS and FSS extracts were biochemically investigated in rats against CCl_4_-induced hepatotoxicity. CCl_4_ injection enlarged rat livers by storing fats inside liver cells [78]. CCl_4_ injection causes cellular leakage and the loss of functional integrity of cell membranes in the liver, as seen by elevated blood enzyme levels (ALT, AST). Administration of FS and FSS aqueous and ethanolic extracts significantly improved the levels of liver enzymes (ALT and AST), which consistently agreed [79]. Similarly, Saxena et al. [80] and Jung et al. [81] have confirmed the effects of the plant-based extract on elevated serum ALT and AST enzymes in rats against CCl_4_-induced oxidative stress. In this regard, the FS and FSS efficiently attenuate liver enzymes owing to their biological substances [80,81].

The catabolite malondialdehyde marker identifies lipid peroxidation and increases the risk of tissue damage caused by the produced ROS [82]. All mammalian cells contain GSH, a non-enzymatic antioxidant. With its oxidized form, GSSG, GSH protects cells from oxidative stress and maintains cellular redox balance by serving as a cofactor for several detoxification enzymes (GPx, GST, and others) [83]. In the same situation, SOD catalyzes the breakdown of two molecules of superoxide anion (*O2) into hydrogen peroxide (H_2_O_2_) and molecular oxygen (O_2_). As a result, the potentially dangerous superoxide anion is rendered less dangerous [82]. One of the most critical indicators of oxidative stress, MDA, is the initial by-product of lipid peroxidation. As measured by the catabolite malondialdehyde, ROS raises the risk of tissue damage and induces lipid peroxidation [84]. SOD, CAT, GPx, and GSH activities were all significantly decreased, while the MDA level was dramatically elevated in previous research involving chronic CCl_4_ i.p. injection [32,85].

As seen in the first part of this study, FS and FSS are rich in phenolic and volatile components, particularly flavonoids, which possess antioxidative capabilities with increased phenolics after sprouting and are thought to have functional and therapeutic benefits [19]. The suggested mechanisms for this amelioration activity may be discussed [32,47,48,50]. In the current study, the predominant phenolics were vanillic acid and Kaempferol, which might work as protective substances [86,87,88]. In addition, GC-MS analysis exhibited two forms of *trans*-Anethole, presenting 61.97%, followed by 11.18% Fenchone. Antioxidant enzymes, including superoxide dismutase (SOD), catalase (CAT), and glutathione peroxidase (GPx), are crucial for neutralizing free radicals [89]. The most efficient treatment of FS or FSS was using a dose of 600 mg kg^−1,^ which recorded an improvement rate of 40.08 and 37.87%, 37.17 and 46.52%, 114.56 and 154.13%, and 66.05 and 69.69% for GSH, DMA, CAT, and SOD when compared to the CCl_4_-group (G2), respectively. Interestingly, treating rats with 600 mg Kg^−1^ FSS extract enhanced the enzymatic defense system significantly better than FS extract compared to normal rats (G1) and CCl_4_-treated rats (G2). According to Rather et al. [13], numerous therapeutic applications for *F. vulgare* have been validated by in vitro and in vivo models, including antifungal, antibacterial, antioxidant, antithrombotic, and hepatoprotective effects. Recently, Samadi-Noshahr et al. [90] reported that FS and *trans*-Anethole could protect the liver against diabetes-induced hepatic injury in rats, probably via hypoglycemic and antioxidant effects. It showed that FS or TA could help manage diabetes complications using 200 and 400 mg Kg^−1^ of FS and 80 mg of *trans*-Anethole, which were close to the concentrations used in our study. Even the induction model was different, as they used STZ-induced diabetes and we used CCl4-induced hepatotoxicity and oxidative stress models. As seen in FSS, 300 mg Kg^−1^ of FSS presented better improvements than the same concentration of FS. However, the aforementioned compounds and numerous plant-based extracts have been shown to have antioxidative and anti-inflammatory effects in rats with hepatic damage [50,90,91].

## 5. Conclusions

This study showed FS’s and FSS’s antioxidative potential in vitro and in vivo. FS and FSS extracts are rich in phenolic and volatile components, mainly antioxidant flavonoids. A phenolic analysis discovered powerful flavonoids in *F. vulgare* sprouts, supporting the plant’s functional and medicinal claims. HPLC examination showed 13 phenolic chemicals (primarily vanillic acid) and six flavonoids (primarily Kaempferol). FS and FSS have different phenolic levels. GC-MS analysis exhibited that the predominant component was Benzene, [1-(2-propenyloxy)-3-butenyl] (*trans*-Anethole) (38.41%), followed by *trans*-Anethole (Benzene, 1-methoxy-4-(2-propenyl)) (23.65%), Fenchone (11.18%), and 1,7-Octadiene, 2-methyl-6-methylene-cyclohexene (7.17%). FSS and FS extracts showed significant oxidative DNA damage prevention. FS and FSS aqueous and ethanolic extracts protect rats from CCl_4_ hepatotoxicity in a dose-dependent manner. The most effective treatment for FS or FSS was 600 mg kg^−1^, which improved ALT, AST, and ALP by 20.77, 24.17, 20.36, and 37.49%, respectively. GSH, DMA, CAT, and SOD improved by 40.08 and 37.87%, 37.17 and 46.52%, 114.56 and 154.13%, and 66.05 and 69.69% in FS and FSS compared to the CCl_4_-group (G2). Treating rats with 600 mg Kg^−1^ FSS extract enhanced the enzymatic defense system significantly better than FS extract compared to normal rats and CCl_4_-treated rats. The protective efficacy could be attributed to the high concentration of phenolics, which can reduce hepatotoxicity complications. Biochemical examinations have confirmed this superior activity. As a result, the findings could aid in explaining the therapeutic efficacy of FS and FSS as functional products. It encouraged us to recommend *F. vulgare* sprout production for combining oxidative stress and hepatotoxicity, as well as being beneficial and profitable for controlling oxidative stress complications. The observed protection is associated with increased phenolics and volatiles in *F. vulgare*, which might accelerate susceptibility to oxidative stress disorders. Including FSS in diets is recommended owing to its functionality, health promotion, and desired prevention capability.

## Figures and Tables

**Table 1:** Quantitative analysis of phenolic compounds in FS, 6th day sprouts, and 9th day sprouts during sprouting at 17 ± 1 °C and 90–93% RH.

Item	No.	Compound	Phenolics (µg g^−1^) *		
			Sprouting Period (day)		
			Raw Fennel Seed	6-Days Sprouts	9-Days Sprouts
Phenolic acids	1	Pyrogallol	-	-	-
	2	Quinol	-	-	-
	3	3-Hydroxytyrosol	-	-	-
	4	Catechol	9.26	40.98	72.26
	5	*p*-Hydroxy benzoic acid	32.25	83.45	32.60
	6	Caffeic acid	26.72	2.40	48.22
	7	Chlorogenic acid	6.79	17.88	71.25
	8	Cinnamic acid	9.38	14.18	18.83
	9	Ellagic acid	25.35	46.20	52.30
	10	Vanillic acid	587.40	105.31	129.08
	11	Ferulic acid	20.01	20.39	48.51
	12	Gallic acid	-	-	-
	13	O-coumaric acid	112.77	9.76	8.44
	14	*p*-coumaric acid	18.46	11.05	19.89
	15	Benzoic acid	30.38	90.74	110.35
	16	Rosmarinic acid	64.41	53.25	124.71
	17	Syringic acid	9.72	10.13	66.08
Flavonoids	1	Catechin	123.46	151.42	151.46
	2	Kaempferol	5913.55	8.24	2357.57
	3	Myricetin	236.93	42.17	166.94
	4	Quercetin	28.71	187.88	192.35
	5	Rutin	423.28	817.03	985.29
	6	Resveratrol	472.19	159.37	402.24
	7	Naringenin	-	-	-

*: Phenolic acids were identified at 284 nm, and flavonoids were identified at 365 nm. The presented data is for duplicate analysis, -: Not detected.

**Table 2:** GC-MS identification and quantification of volatile compounds in *F. vulgare* seeds and their 9-day prepared sprouts at 17 ± 1 °C and 90–93% RH, (mean ± SE), *n* = 3.

No.	R_t_	Compound	MW	Chemical Formula	Peak Area %		
					Raw Fennel Seed	6-Days Sprouts	9-Days Sprouts
1	6.50	α-Pinene	136	C_10_H_16_	0.44	1.14	-
2	7.63	(E)-3-Propylidenecyclopentene	108	C_8_H_12_	0.27	-	-
3	8.21	α-Myrcene	136	C_10_H_16_	0.14	-	-
4	9.21	1,7-Octadiene, 2-methyl-6-methylene-Cyclohexene	136	C_10_H_16_	7.17	-	3.32
5	9.55	α-Pinene	136	C_10_H_16_	0.17	0.19	0.1
6	10.13	ç-Terpinene	136	C_10_H_16_	0.1	-	-
7	10.95	Fenchone	152	C_10_H_16_O	11.18	14.19	7.42
8	11.24	2,6,10-trimethyl-tridecane	226	C_16_H_34_	0.14	0.13	-
9	11.96	*cis*-Verbenol	152	C_10_H_16_O	0.07	-	-
10	12.28	*cis*-Limonene oxide	152	C_10_H_16_O	0.08	0.1	-
11	12.41	*trans*-Limonene oxide	152	C_10_H_16_O	0.09	0.1	0.06
12	12.54	Naphthalene, decahydro-2-methyl	152	C_11_H_20_	0.19	0.2	0.16
13	14.30	*trans*-Anethole (Benzene, 1-methoxy-4-(2-propenyl))	148	C_10_H_12_O	23.65	33.47	22.89
14	15.10	Exobornyl acetate	168	C_12_H_20_O_2_	0.1	-	0.09
15	15.45	2-Cyclohexen-1-one, 2-methyl-5-(1-methylethenyl)	150	C_10_H_14_O	0.13	-	-
16	15.82	4-Methoxybenzaldehyde (4-Anisaldehyde)	136	C_8_H_8_O_2_	1.45	2.38	2.32
17	15.87	Hydrazine, phenyl, monohydrochloride	144	C_6_H_9_ClN_2_	1.81		
18	16.77	Benzene, [1-(2-propenyloxy)-3-butenyl] (trans-Anethole)	188	C13H16O	38.41	19.01	42.32
19	19.20	(4R,5S)-1-Ethoxy4methoxy-5-[(4-methoxybenzyl)oxy]hept-1-yn-6-ene	304	C_18_H_24_O_4_	0.43	-	0.18
20	20.89	(3R,3aR)-1,2,3,4,5,6-hexahydro-3-methyl-3aH-indene-3a-carbaldehyde	196	C_11_H_16_O	0.11	-	0.05
21	21.17	*m*-Anisic acid, 3,4-dichlorophenyl ester	296	C_14_H_10_Cl_2_O_3_	0.1	-	-
22	23.35	1,2-Dimethyl-3-nitro-4-nitrosobenzene	180	C_8_H_8_N_2_O_3_	0.3	-	-
23	23.47	Trans-2-Tridecenal	196	C_13_H_24_O	0.24	-	0.14
24	23.87	Benzeneacetic acid, α-hydroxy-4-methoxy	182	C_9_H_10_O_4_	1.65	3.18	3.34
25	24.03	z-isomer, 2-(2-hydroxyethylidene)-3-methoxynorbornane	168	C_10_H_16_O_2_	0.21	0.52	0.46
26	25.08	1-(4-Methoxyphenyl) propan-1-ol	166	C_10_H_14_O_2_	0.12	-	-
27	25.76	Ethanone, 1-(1-hydroxy-2,6,6-trimethyl-2,4-cyclohexadien-1-yl)	180	C_11_H_16_O_2_	0.26	-	-
28	26.13	2-Propanone, 1-(4-hydroxy-3-methoxyphenyl)	180	C_10_H_12_O_3_	1.8	0.98	0.51
29	26.55	(4-Methoxy-phenyl)-(2-nitrocyclohexyl)-methanol	265	C_14_H_19_NO_4_	0.19	0.11	0.52
30	26.87	4-Methoxyphenylethyleneglycol	168	C_9_H_12_O_3_	1.86	-	-
31	32.21	Pentamethyl Pentaphenyl Cyclopentasiloxane	680	C_35_H_40_O_5_Si_5_	0.13	-	-
32	34.08	9,12-Octadecadienoic acid (Z,Z)-, methyl ester	294	C_19_H_34_O_2_	0.11	0.23	0.18
33	34.2	6-Octadecenoic acid, methyl ester	296	C_19_H_36_O_2_	0.23	0.59	0.89
34	35.35	9-Octadecenoic acid (Z), ethyl ester	310	C_20_H_38_O_2_	0.09	0.09	0.75
35	35.57	4,4′-Di(3-butenyl)-2,2′-bipyridine	264	C_18_H_20_N_2_	3.12	-	-
36	37.12	Benzaldehyde N,N-dimethylhydrazone	148	C_9_H_12_N_2_	0.09	0.09	-
37	37.47	Z-7-Pentadecenol	226	C_15_H_30_O	0.08	0.12	-
38	39.63	Cyclohexane,1,1′-dodecylidenebis[4-methyl]	362	C_26_H_50_	0.11	-	-
39	39.83	1-Ethyl-2-formyl-9-methy-l-4-oxo1,2,3,4-tetrahydr-o-α-carboline	256	C_15_H_16_N_2_O_2_	0.2	0.2	0.13
40	40.04	6-Methyl-6-(3′-isopropeny-l-2′-methyl-cycloprop-1′-en1′-yl)-2-heptanol	222	C_15_H_26_O	0.15	-	-
41	40.36	9-Octadecenoic acid (Z)	282	C_18_H_34_O_2_	0.29	0.22	0.24
42	40.61	Ethanol, 2-ethoxy, acetate (6-Tridecene)	139	C_9_H_15_D_2_N	0.08	-	-
43	41.7	*Tert*-Butyl ester of 3,4-Dimethyl-5-(2-nitroethyl)-pyrrol-2-carboxylic acid	268	C_13_H_20_N_2_O_4_	0.14	-	-
45	41.9	1-(*p*-hydroxy tolyl) propan-1-ol (impure)	166	C_10_H_14_O_2_	0.08	0.17	-
46	42.46	Ethanone, 2-hydroxy-1,2-bis(4-methoxyphenyl)	272	C_16_H_16_O_4_	0.19	-	-
47	42.8	Ethanone, 2-hydroxy-1,2-bis(4-methoxyphenyl)	272	C_16_H_16_O_4_	0.17	-	-
48	43.54	Nonacosane	408	C_29_H_60_	0.08	0.15	0.08
49	46.28	Docosane	310	C_22_H_46_	0.1	0.11	-
50	48.79	2-Diisobutylcarbamoyl-cyclohexane carboxylic acid, decyl ester	423	C_26_H_49_NO_3_	1.57	-	-
51	51.15	{[3E)-2-[(Dimethylcarbamoyl)methyl]-3-ethylidene-13,17-bis[2′(methoxycarbonyl)ethyl]2,7,12,18-tetramethyl-2,3dihydroporphytinato]}vzinc (II)	713	C_38_H_43_N_5_O_5_Zn	0.08	-	-
52	-	unknown	-	-	0.05	0.17	0.01
Total					100		

## Data Availability

Data is contained within the article.

## Supplementary Materials

The following supporting information can be downloaded at: https://www.mdpi.com/article/10.3390/antiox11122318/s1, Figure S1: HPLC chromatograms of fennel seeds and their sprouts during sprouting for 15 days at 17 ± 1 °C and 90–93% RH. (**A**): Raw fennel seeds, (**B**): 6-days sprouts, and (**C**): 9-days sprouts.

## Abbreviations

AOA: Antioxidant activity; DPPH: 1,1-diphenyl-2-picryl hydrazine; dw: Dry weight; FS: Fennel seeds; FSS: Fenne; GA: Gallic acid; GAE: Gallic acid equivalent; GC-MS: Gas chromatography-Mass spectroscopy; HPLC: High-performance liquid chromatography; QE: Quercetin equivalent; RSA: Radical scavenging activity; SE: Standard error; TBA, Thiobarbituric acid; TE: Trolox equivalents; TF: Total flavonoids; TFL: Total flavonols.
