# Supplementary material for: Phenolics and Volatile Compounds of Fennel (Foeniculum vulgare) Seeds and Their Sprouts Prevent Oxidative DNA Damage and Ameliorates CCl4-Induced Hepatotoxicity and Oxidative Stress in Rats
Source: Antioxidants (Basel). 2022 Nov 23;11(12):2318. doi: 10.3390/antiox11122318 (PMC9774655; doi:10.3390/antiox11122318)
Supplement: Supplementary file 1 [file antioxidants-11-02318-s001.zip › antioxidants-1954008-supplementary.pdf]

antioxidants

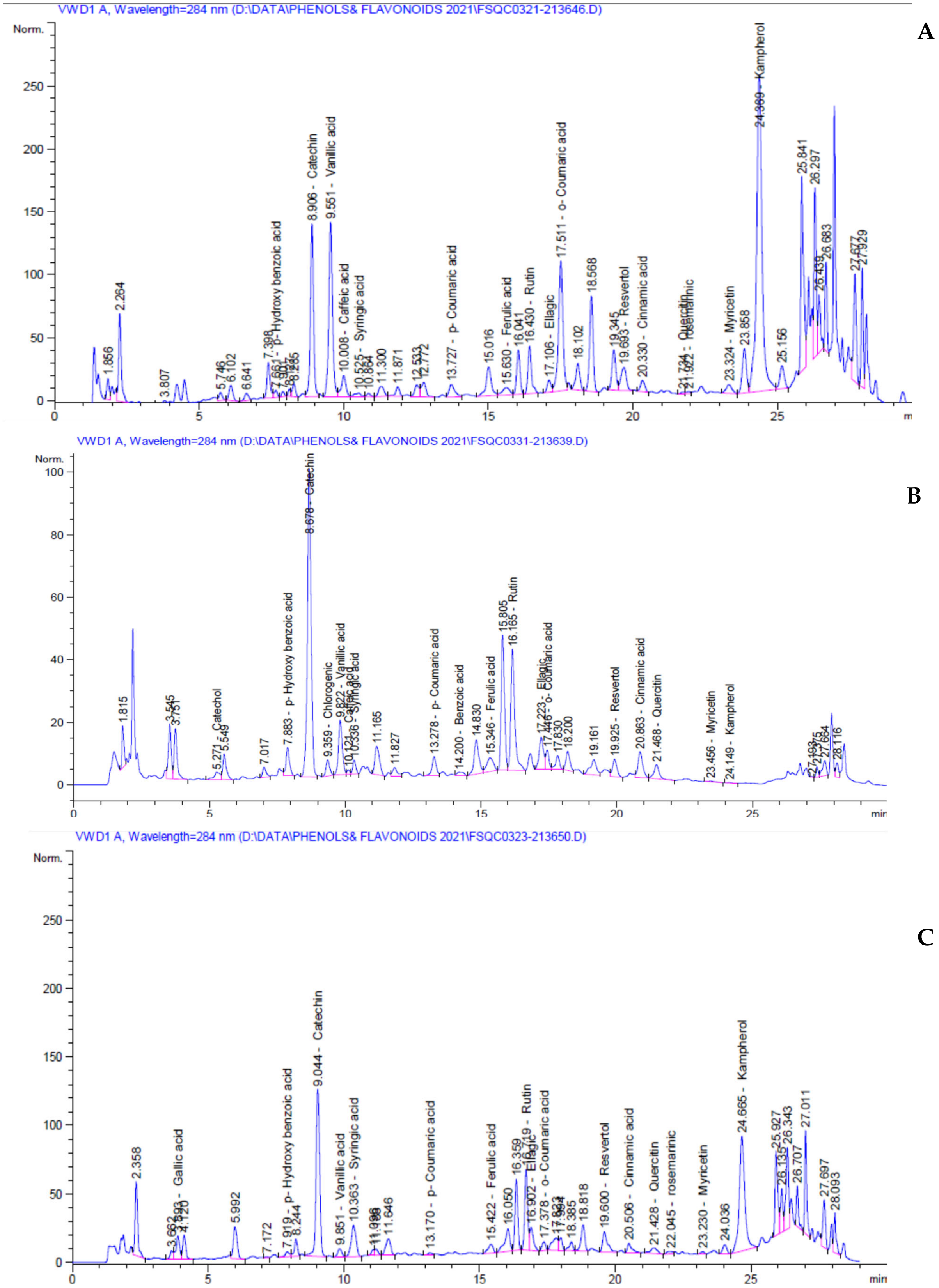

Figure S1. HPLC chromatograms of fennel seeds and their sprouts during sprouting for 15 days at 17 ± 1°C and 90–93% RH. (**A**): Raw fennel seeds, (**B**): 6-days sprouts, and (**C**): 9-days sprouts. .
